# Maternal investment, life-history trajectory of the off-spring and cardiovascular disease risk in Emirati females in the United Arab Emirates

**DOI:** 10.1186/s12889-021-11182-0

**Published:** 2021-06-27

**Authors:** Rola Al Ghali, Linda Smail, Maryam Muqbel, Dalia Haroun

**Affiliations:** 1 Department of Health Sciences, Zayed University, College of Natural and Health Sciences, Dubai, United Arab Emirates; 2 Department of Mathematics and Statistics, Zayed University, College of Natural and Health Sciences, Dubai, United Arab Emirates; 3 Department of Medicine, Imperial College, London, UK

**Keywords:** Adiposity, low birth weight, Maternal investment, Life-history trajectory, Cardiovascular disease

## Abstract

**Background:**

Variations in cardiovascular disease risk (CVD) are suggested to be partly influenced by factors that affect prenatal growth patterns and outcomes, namely degree of maternal investment (proxied by birth weight and gestational age). Using the life history trajectory model, this study investigates whether maternal investment in early prenatal life associates with menarcheal age and whether maternal investment affects CVD risk in adulthood and predicts adult size and adiposity levels.

**Methods:**

A cross-sectional study was conducted among 94 healthy Emirati females. Birth weight, gestational age and menarcheal age were obtained. Anthropometrical measurements, body composition analysis, and blood pressure values were collected. Regression analyses were conducted to establish associations.

**Results:**

There was no association between birth weight standard deviation score (SDS) and age at menarche. When investigating the associations of birth weight SDS and age at menarche with growth indices, it was found that only birth weight was positively and significantly associated with both height (*β* = 1.342 cm, 95% CI (0.12, 2.57), *p* = 0.032) and leg length (*β* = 0.968 cm, 95% CI (0.08, 1.86), *p* = 0.034). Menarcheal age was significantly and inversely associated with fat mass index (FMI) (*β* = − 0.080 cm, 95% CI (− 0.13, − 0.03), *p* = 0.002), but not with waist circumference and fat free mass index (FFMI) (*p* > 0.05). Birth weight SDS was positively and significantly associated with waist circumference (*β* = 0.035 cm, 95% CI (0.01, 0.06), *p* = 0.009), FMI (*β* = 0.087 cm, 95% CI (0.01, 0.16), *p* = 0.027), and FFMI (*β* = 0.485 cm, 95% CI (0.17, 0.80), *p* = 0.003). Birth weight SDS was not significantly associated with either systolic blood pressure (SBP) or diastolic blood pressure (DBP) (*p* > 0.05). However, FMI, waist circumference, and FFMI were positively and significantly associated with SBP. Regarding DBP, the relationship was negatively and significantly associated with only FFMI (*β* = − 1.6111 kg/m2, 95% CI (− 2.63, − 0.60), *p* = 0.002).

**Conclusion:**

Although the results do not fully support that Emirati females fast-life history is associated with increased chronic disease risk, the data does suggest a link between restricted fetal growth in response to low maternal investment and metabolic and reproductive health.

**Supplementary Information:**

The online version contains supplementary material available at 10.1186/s12889-021-11182-0.

## Background

Current World Health organization (WHO) statistics have reported Cardiovascular Disease (CVD) to be the leading cause of mortality worldwide with an estimation of 17.9 million deaths each year [1]. In the United Arab Emirates (UAE), the burden of CVD is no different, where a recent report estimates that 40% of total deaths are linked to CVD [2]. The aetiology of heart disease is complex and multifactorial [3], however there are various factors that have been implicated in increasing disease risk. Lifestyle risk factors include smoking, physical inactivity, obesity (with strong emphasis on central/visceral adiposity) and alcohol intake [4]. These behavioral risk factors are often manifested into one or more of the following: high blood pressure (BP), insulin resistance, diabetes, increased blood lipids, and even kidney dysfunction [5, 6].

Hypertension is a highly prevalent condition that affects up to 1.13 billion adults all over the world and is highly incriminated in increased risk of CVD amongst other chronic conditions [7]. Normal readings of both systolic blood pressure (SBP) and diastolic blood pressure (DBP) are essential to optimal functioning of all vital organs. However, studies have demonstrated that SBP is possibly more significant as an independent predictor of risk for stroke, heart failure and other coronary events [8]. Numerous factors contribute to the development of hypertension, including intrauterine growth restriction (IUGR), which has been linked to high BP in adulthood. This could be attributed to the effect that restricted growth has on the number of nephrons an individual develops, a number that is set at birth [5, 9]. Mounting evidence has found that retarded growth in utero is significantly associated with a reduction in nephron number, which in turn is a risk factor for the development of hypertension. Animal studies have shown that rats subjected to growth retardation by restricting maternal protein intake presented with a nephron deficit in comparison to the control group [10]. Therefore, low birth weight for gestational age can be used as a marker to link intrauterine growth and the effect maternal investment potentially had on the offspring’s capacity for maintaining metabolic homeostasis [5]. A multitude of studies have demonstrated the significant links between low birth weight and elevated BP in adults [5, 11, 12]. In 2020, Aye et al. [13], reported that maternal hypertension was associated with ‘both prenatal and postnatal differences in cardiac development, with right ventricular changes proportional to the severity of the pregnancy disorder’. Yet, long term effect and relationship to CVD is still not fully explored as more work is needed to determine if these changes persist into adulthood and whether they impact the physical, respiratory, and cardiac functions [13].

Adiposity-related health consequences are well known and documented, and high adiposity levels have been linked to numerous chronic diseases including CVD [14]. Body Mass Index (BMI) continues to be used as a marker for obesity levels and disease risk, however it is just as important to take into account body fat distribution due to the fact that excess central adiposity is detrimental to health and is highly correlated with a host of metabolic diseases including increased CVD risk [14]. Because precise measurement of central fat is expensive and requires radiological imaging, waist circumference is accepted and often used as a surrogate marker instead [15]. Evidence suggests that the intrauterine environment as well as the early infancy period could be responsible for shaping body composition in later life, where several studies have found an inverse association between birth weight and greater BMI as well as higher abdominal obesity levels (larger waist circumference) [16–18]. A cohort study of indigenous Australians found that those with the highest CVD risk were both in the highest waist circumference quartile group and of low birth weight [19]. Boscaini & Pellanda [20] revealed that high birth weight individuals had higher BMI and waist circumference. This implies that the relationship is much more complex and our understanding of it needs further exploration [21]. Sex is another factor added to the birth weight and adiposity association. Rockenbach et al. [22] examined sex-specific relationship of birth weight with measures of adiposity. Results in men showed that low birth weight was linked with lower adiposity while low birth weight in females showed greater adiposity [22].

The risk factors explored above are only able to predict disease risk up to a limited point. So far, the lifestyle model has only been successful in partly explaining CVD risk by exploring lifestyle interactions with underlying genetic influences [23]. Other theories were developed in an attempt to explain the complex nature of heart disease aetiology to help understand how prenatal period influences can bridge the gap in our understanding of both CVD aetiology [24] and prenatal associations with chronic disease susceptibility [25].

Hales and Barker [26], in accordance with earlier observations by Forsdahl [27], proposed one of the earliest models explaining this link. They found that CVD mortality rates were recorded to be highest in areas of poverty that were exposed to later prosperity. If later affluence and nutritional overabundance in childhood and adulthood was not experienced, the risk did not appear to be increased [27]. This implied that energy insufficiency in-utero could affect the ability to manage nutritional affluence (excessive intake of energy) later on in life [5]. They suggested that CVD and other chronic diseases are ‘programmed’ during critical windows in fetal life and even into early infancy. This hypothesis was termed ‘fetal origins of adult disease’ [28]. Research has found that suboptimal nutrition in-utero, particularly halfway through the gestational period, increases CVD risk in adulthood by “the programming of blood pressure, cholesterol metabolism, blood coagulation, and hormonal settings” [24]. Specifically, arteriosclerotic heart disease was linked to an increase in serum cholesterol levels in those who grew up in least affluent households and were exposed to a high fat diet [27].

Although past studies have shown that biological programming in prenatal life as a response to nutritional affluence or lack of is an important factor to consider, it is important to also take into account how lifestyle measures later on in life affect the degree of disease manifestation. To address this issue, Wells et al. [5] went on to propose a ‘capacity-load’ model for risk of disease. This builds on the original ‘thrifty phenotype’ hypothesis by Hales and Barker [26] and incorporates two different variables: metabolic capacity and metabolic load. The former refers to the ability to maintain homeostasis throughout adulthood and stems from prenatal growth (birth weight and gestational age). The latter is defined as various stresses that strain cardio-metabolic function, such as obesity, smoking, sedentary lifestyle and inflammation.

During critical windows in gestation, “epigenetic processes are thought to alter gene expression in the fetus based on maternal environmental cues” [29] in order to best prepare the fetus for the extra uterine environment. A ‘mismatch’ in the predicted environment due to exposure to nutritional affluence post birth is proposed to increase disease risk. Therefore, the phenotype developed during those windows of under nutrition in order to aid in survival, results in lower ability to tolerate metabolic load later on in life [30]. In this context, diminished metabolic capacity becomes an issue only when metabolic load is intensified [30]. This explains why individuals who are not exposed to nutritional affluence after birth do not appear to have heightened metabolic risk even if exposed to malnutrition in the uterine environment [5]. It also explains why those born with a lower birth weight (lower metabolic capacity) and become large (high metabolic load) exhibit higher risk of CVD [5, 31, 32]. Wells et al. [5] also suggested that life-history theory could explain why individuals have varying ranges of load and capacity. This theory proposes that individuals must allocate their energy resources in a way that maximizes fitness. They can either invest in characteristics that “affect the age-schedule of mortality; or in traits that affect the age-schedule of fertility” [33]. For example, investing in reproductive fitness (higher adiposity stores) could reduce investment in growth. This theory could be used to justify why energy allocation during periods of nutritional insufficiency during the life course is directed towards reproductive functions instead of maintenance ones [34]. According to this evolutionary theory, individuals who were recipients of diminished maternal investment (restricted nutritional environment in utero proxied by lower birth weight and shorter gestational period) can be said to have a fast-paced life-history strategy [5]. This translates to earlier maturation in the form of earlier menarche as well as a shorter stature. This is suggested to lead to a reduced metabolic capacity, and a high metabolic load that increases CVD risk. So, lower maternal investment  faster pace of life history  lower adult height, higher energy stores for reproduction and lower metabolic health. In other words, low birth  weight earlier maturation + shorter height  later increased adiposity. The opposite holds true for those receiving high maternal investment and optimal nutrition in fetal life [5]. In 2018, Wells proposed the relative partitioning concept of maternal investment between fetal life and infancy. This analytical framework suggests that low fetal weight gain diminishes the energy needed for growth and maintenance, and if continued, growth postnatally will continue to be constrained. This leads to earlier menarche and reproductive maturation, small adult size, elevated fat stores. ‘Through the partitioning described above, the combination of short adult stature and high fatness favour the same trade-offs emerging in the next generation’ [35]. Bleil et al. [36] reported that younger menarcheal age was related to greater CVD risk. A study conducted in the UK found that less than optimal maternal investment led to faster life histories which were marked by earlier menarcheal age, shorter stature and higher adult adiposity levels [5]. Another study of Indian girls, who moved to Sweden, found that those with the most stunted growth had earlier menarche as well [37]. Maternal investment is defined as a combination of two elements: maternal resources at conception (proxied by her size and nutritional status) and the resources provided by her to offspring though pregnancy and then lactation, stemming from her nutritional intake [31]. For the purpose of this study, birth weight SDS, computed from birth weight and gestational age, is used as a marker to reflect degree of maternal investment; this being consistent with earlier studies [5].

Low birth weight, which is an indicator of poor fetal growth and low maternal investment, has been found to result in lower fat free mass (FFM) in adulthood [38–40]. This could partly explain why lower birth weight is linked to an increased risk of CVD, since a lower muscle mass could lead to impaired glucose regulation [38]. This is due to the fact that muscles play a critical role in the homeostasis of blood sugar, as 70–90% of the glucose is taken up by the muscles [41] making the muscles ‘the primary tissue contributing to whole-body insulin-mediated glucose disposal’ [42]. Therefore, lower FFM could lead to development of insulin resistance, which is an impairment that contributes heavily to CVD development in adulthood [38]. Different theories have been proposed in an attempt to explain the link between prenatal life factors and occurrence of CVD. However, further studies still need to be carried out in order to establish whether early malnutrition in-utero negatively influences metabolic competence and organ physiology [31].

### Aim of study

To the best of our knowledge, this study is the first one of its kind in the UAE. It investigates how maternal investment shapes the life history pace of the offspring. This will offer us a chance to study the extent that maternal investment influences life history pace and generates the trade-off between maturation/adiposity versus growth/homeostatic maintenance. It then looks at whether this trade-off increases risk of CVD in adulthood.

### Hypotheses

In healthy women from the UAE, based on the interpretations of the life history theoretical framework, we hypothesized that:

- Those who were on the receiving end of poor maternal investment (characterized by lower birth weight standard deviation score (SDS)) during the fetal period would develop earlier maturation (proxied by earlier age of menarche).
- Lower maternal investment, coupled with an earlier maturational timing (proxied by earlier age of menarche), would then lead to reduced growth (proxied by shorter height, and leg length).
- Lower maternal investment, coupled with an earlier maturational timing (proxied by lower birth weight SDS and earlier age at menarche), would then lead to elevated CVD risk (proxied by greater waist circumference, higher fat mass index (FMI) and lower fat free mass index (FFMI).
- Reduction in metabolic capacity (proxied by low birth weight) in addition to a high metabolic load (proxied by elevated adult adiposity and lower FFMI) would lead to higher risk of CVD (proxied by elevated BP).

## Methods

### Subjects

This was a cross-sectional study that involved 94 females aged between 17 and 27 years with a gestational age exceeding 37 weeks. The study used convenience sampling method for selecting healthy, Emirati females enrolled in various governmental (public) universities in the UAE. The consent of participants was obtained, and data collection was carried out in the body composition laboratory at Zayed University, Dubai-UAE. The study was reviewed and received full ethical clearance by the Zayed University Research Ethics Committee (ZU16_016_F). Exclusion criteria included current smokers, twins, pregnant or lactating females, those with metal parts in their body (as this contraindicates the use of the dual energy X-ray absorptiometry (DXA), those who reported a weight gain or loss of more than 3 kg in the 3 months leading up to the screening, and those with medical conditions known to impact body composition or metabolism such as diabetics, renal patients, hypertensives and females with hormonal imbalances.

### Demographics, maternal investment & maturational timing

A structured questionnaire was used to obtain personal data such as date of birth, sex, ethnicity, marital status, birth order, gestational age, weight at birth, menarcheal age, medical history, list of medications taken if any, and family history of disease (Additional file 1). To obtain the maternal data such as birth weight, gestational age, and infant feeding mode/duration, participants were asked to contact their mothers during the session. Breastfeeding has been proven to be protective against overweight and obesity [43]. Controlling for breast feeding reduces the confounding factors that may contribute to weight changes, hence affecting body composition and BP. All this information was obtained via recall, as verifying by other records was not possible. Birth weight SDS were computed by adjusting for gestational age. Birth weight SDS was used to assess maternal nutritional investment. Age at menarche was used as a marker for timing of reproductive maturation, whereas FFMI, height and leg length were seen as markers for growth outcomes, and FMI as the potential to invest in reproduction, as fat mass reflects the energy reserves for reproduction [5]. To assess potential risk of CVD, blood pressure and waist circumference were used [5].

### Anthropometry and body composition

Weight was obtained to the nearest 0.01 kg. A stadiometer was used to measure both the standing and the sitting height (reported to the nearest 0.1 cm). The difference between sitting and standing height was then calculated to represent leg length [44]. Both sitting height and leg length were measured in order to gain a full analysis of growth as stature results from changes in length of upper and lower body segments with age. Legs grow relatively faster than other body segments; hence, Leg length is considered a good indicator of the ‘quality of the environment for growth during infancy, childhood and the juvenile years of development’ [45]. In this paper, similar to other studies, leg length was used to correlate between fetal growth, sexual maturation and lower limb growth [5, 46].

Waist circumference was measured at the narrowest position using a nonelastic flexible tape, taken to the nearest 0.1 cm over light clothing [47]. BMI was calculated as weight (kg)/height (m)^2^. WHO classifies overweight as BMI values between 25 and 29.9 kg/m2, and obesity ≤30 kg/m2 [48]. The use of BMI as a risk assessment tool may not always be accurate [49]; therefore, it has been suggested that body composition may be more accurately evaluated by assessing both fat mass and FFM [50]. These values were acquired by DXA. However, it has been pointed out that fat mass and FFM are not adjusted for body size and that height needs to be considered in order to better predict risk of CVD, therefore a FMI and a fat free mass index FFMI have been proposed [51]. FMI is calculated as fat mass (kg)/height (m)^2^ and FFMI = fat free mass (kg)/height (m)^2^.

### Blood pressure

BP was measured using an automatic monitor (Omron, 2016) whilst the subject was seated. The reading was taken three times (a few minutes apart) and the average of the 3 measurements was used in analysis. The American Heart Association (AHA) classifies hypertension as > 140 mmHg systolic and > 90 mmHg diastolic [52].

### Statistics

Data was analyzed using Statistical Package for the Social Sciences (SPSS) software Version 27. Birth weight was adjusted for gestational age, where birth weight SDS were calculated using the LMS method [53]. Linear regression analysis was conducted to investigate: whether maturational timing (proxied by age at menarche) was predicted by degree of maternal investment (gestational age and birth weight SDS); whether growth (height and leg length) and adult adiposity (FMI, waist circumference) were predicted by degree of maternal investment (birth weight SDS) as well as maturational timing (proxied by age at menarche). Linear regression analysis was also used to investigate whether CVD risk (BP, waist circumference, FFMI and FMI) was predicted by degree of maternal investment (birth weight SDS). Adult adiposity levels were also used to predict BP in addition to maternal investment predictors. Linearity and homoscedasticity assumptions for all independent variables were met except for waist circumference, and FMI. These were natural log-transformed, and the regression was then re-fitted accordingly. Statistical tests with *p*–values **<** 0.05 were considered statistically significant.

## Results

### Description of study subject characteristics

A total of 94 participants were recruited in this study. Due to 6 missing measurements, the analysis for sitting height and leg length was done on 88. The mean age of the participants was 19.9 years, while the mean birth weight was found to be 3.1 kg. All those analyzed were born to term according to conventional criteria (> 37 weeks gestation). Regarding BMI, 19 participants (20.2%) were found to be overweight and 8 (8.5%) were obese, and 12.8% had a waist circumference measurement equal to or above 80 cm, which classifies them as having an increased risk of metabolic complications (Disease risk for type 2 diabetes, hypertension, and CVD) according to WHO classifications [47]. The mean SBP for participants was 109.1 mmHg, and the DBP was 71.6 mmHg, both mean results falling within the ‘normal’ blood pressure boundaries in accordance with classifications of the AHA [52]. Most of the participants (73.4%) had a normal BP, 11.7% had an elevated BP, 12.8% has stage 1 hypertension, while 2.1% had stage 2 hypertension. Descriptive statistics for all participants included in analysis are displayed in Table 1. In addition, the majority (88%) had been breastfed with a median duration of 12 months.

**Table 1:** Description of study subject characteristics

	N	Minimum	Maximum	Mean ± SD
**Development**				
Birth weight (kg)	94	2	4	3.1 ± 0.5
Gestational age (weeks)	94	37	42	39.5 ± 1.04
Birth Weight SDS	94	−2.57	1.73	−0.033 ± 1.001
Age at menarche (years)	94	9	16	12.5 ± 1.6
**Adulthood**				
Age (years)	94	17	27	19.7 ± 1.9
Weight (kg)	94	36.1	102.9	57.7 ± 12.6
Height (cm)	94	144.1	176	158.8 ± 6.0
Sitting height (cm)	88	74.8	92.1	83.1 ± 3.1
Leg length (cm)	88	66	84.9	76.0 ± 4.1
Waist circumference (cm)	94	54	102.2	68.8 ± 9.4
Fat (%)	94	23.9	52.9	39.2 ± 6.5
Fat mass (kg)	94	8.9	68.6	22.3 ± 10.0
FFM (kg)	94	23.7	47.1	33.8 ± 5.0
FFMI (kg/m^2^)	94	9.7	17.8	13.4 ± 1.6
FMI (kg/m^2^)	94	3.8	26	9.2 ± 4.0
BMI (kg/m^2^)	94	15.8	38.9	22.8 ± 4.4
SBP (mmHg)	94	73.7	139	109.1 ± 10.5
DBP (mmHg)	94	49.7	97	71.6 ± 7.9

### Associations of maternal investment (proxied by birth weight SDS) with maturational timing (proxied by age at menarche)

Table 2 illustrates the regression model exploring the relationship between maternal investment (birth weight SDS) and maturational timing (age at menarche). A negative association was found between birth weight SDS and age at menarche; however, this was not statistically significant (*β*= − 0.184 years, 95% CI (− 0.51, 0.14), *p* = 0.261).

**Table 2:** Maternal investment predictor of age at menarche

Outcome	Predictors	*β*	SE	*p*-value	R^2^
Age at Menarche (year)	Constant	12.462	0.162	< 0.001	0.014
	Birth Weight SDS	-0.184	0.163	0.261	

*β* change in the outcome resulting from a one-unit change in the predictor value, *SE* Standard Error, *p* significance < 0.05, *R*^*2*^ % of variance explained by the model

### Associations of maternal investment and menarcheal age with growth (proxied by height and leg length)

When investigating the associations of birth weight SDS and age at menarche with growth indices (height and leg length), it was found that birth weight SDS was positively and significantly associated with both height (*β* = 1.342 cm, 95% CI (0.12, 2.57), *p* = 0.032) and leg length (*β* = 0.968 cm, 95% CI (0.08, 1.86), *p* = 0.034). Age at menarche, on the other hand, did not show the same relationship with both growth indices (Table 3). When we looked at the effect size, we noticed that highest birth weight SDS is predicted to be associated with 5.77 cm increase in height compared to the lowest birth weight SDS. This is not an insignificant amount, though it is within the sample’s standard deviation in height which is 6 cm. From Another side, the highest birth weight SDS is predicted to be associated with 4.16 cm increase in leg length compared to the lowest birth weight SDS. This is not an insignificant amount, though it is slightly higher than the sample’s standard deviation in leg length which is 4.1 cm.

**Table 3:** Developmental predictors of adult stature

Outcome	Predictors	*β*	SE	*p*-value	R^2^
Height (cm)	Constant	152.370	4.940	< 0.001	0.061
	Birth Weight SDS	1.342	0.617	0.032	
	Age at menarche (year)	0.522	0.393	0.188	
Leg Length (cm)	Constant	73.258	3.412	< 0.001	0.030
	Birth Weight SDS	0.968	0.449	0.034	
	Age at menarche (year)	0.222	0.270	0.415	

*β* change in the outcome resulting from a one-unit change in the predictor value, *SE* Standard Error, *p* significance < 0.05, *R*^*2*^ % of variance explained by the model.

### Associations of maternal investment and maturational timing (proxied by age at menarche) with CVD risk (proxied by waist circumference, FMI and FFMI)

Table 4 displays the associations of birth weight SDS and age at menarche with adult adipose indices (FMI and waist circumference). Menarcheal age was significantly and inversely associated with FMI (*β* = − 0.080 cm, 95% CI (− 0.13, − 0.03), *p* = 0.002), indicating higher adult adiposity with earlier puberty. Menarcheal age was not significantly associated with waist circumference and FFMI (*p* > 0.05). On the other hand, birth weight SDS was positively and significantly associated with waist circumference (*β* = 0.035 cm, 95% CI (0.01, 0.06), *p* = 0.009), FMI (*β* = 0.087 cm, 95% CI (0.01, 0.16), *p* = 0.027), and FFMI (*β* = 0.485 cm, 95% CI (0.17, 0.80), *p* = 0.003). The highest birth weight SDS is predicted to be associated with 1.16 cm increase in waist circumference compared to the lowest birth weight SDS. This is not an insignificant amount, though it is within the sample’s standard deviation in waist circumference which is 9.4 cm. When age was accounted for in the models, it showed significant result only with FFMI (*β* = 0.217 years, 95% CI (0.05, 0.38), *p* = 0.011). In this same model, age at menarche showed significant association with FFMI as well (*β* = − 0.222 years, 95% CI (− 0.42, − 0.02), *p* = 0.029), where *β*-coefficient of age at menarche dropped from − 0.181 (95% CI -0.38, 0.02) to − 0.222 (95% CI -0.42, − 0.02) with a change in *p*-value to < 0.05. When duration of breast feeding (months) was introduced to the models, positive but not significant associations were found (*p* > 0.05) with waist circumference, FMI and FFMI.

**Table 4:** Developmental predictors of maternal investment, age at menarche, adult adiposity and FFMI indices

Outcome	Predictors	*β*	SE	*p*-value	R^2^
Ln Waist Circumference (cm)	Constant	4.368	0.105	< 0.001	0.100
	Birth Weight SDS	0.035	0.013	0.009	
	Age at menarche (year)	−0.012	0.008	0.171	
Ln FMI (kg/m^2^)	Constant	3.145	0.309	< 0.0001	0.162
	Birth Weight SDS	0.087	0.039	0.027	
	Age at menarche (year)	−0.080	0.025	0.002	
FFMI (kg/m^2^)	Constant	15.650	1.281	< 0.001	0.132
	Birth Weight SDS	0.485	0.160	0.003	
	Age at menarche (year)	−0.181	0.102	0.079	
Ln Waist Circumference (cm)	Constant	4.566	0.161	< 0.001	0.192
	Birth Weight SDS	0.038	0.013	0.005	
	Age at menarche (year)	−0.009	0.008	0.264	
	Breast feeding (months)	−0.011	0.007	0.110	
FFMI (kg/m^2^)	Constant	11.879	1.915	< 0.001	0.192
	Birth Weight SDS	0.431	0.157	0.007	
	Age at menarche (year)	−0.222	0.100	0.029	
	Age (year)	0.217	0.084	0.011	

*β* change in the outcome resulting from a one-unit change in the predictor value, *SE* Standard Error, *p* significance < 0.05, *R*^*2*^ % of variance explained by the model

### Associations of maternal investment (low birth weight) and adult tissue masses (metabolic load) with CVD risk (proxied by systolic and diastolic blood pressure)

Table 5 assesses the associations of birth weight SDS (maternal investment) and adult body composition (FMI, FFMI, and waist circumference) with blood pressure regulation (marker of CVD risk). Birth weight SDS was not found to be significantly associated with either SBP or DBP in any of the models described below (*p* > 0.05). However, all indices of adult adiposity (FMI and waist circumference) and FFMI were positively and significantly associated with SBP. Regarding DBP, the relationship was only negatively and significantly associated with FFMI (*β* = − 1.6111 kg/m2, 95% CI (− 2.63, − 0.60), *p* = 0.002). When age was introduced to the BP regression models, no significant changes in the associations were found.

**Table 5:** Capacity-load models of systolic and diastolic blood pressure (SBP and DBP)

Outcome	Predictors	*β*	SE	*p*-value	R^2^
SBP (mmHg)					
Model 1	Constant	89.437	5.733	< 0.001	0.152
	Birth Weight SDS	1.124	1.052	0.288	
	Ln FMI (kg/m^2^)	9.177	2.630	0.001	
Model 2	Constant	−36.36	32.85	0.271	0.240
	Birth Weight SDS	1.370	1.020	0.183	
	Ln Waist Circumference (cm)	34.440	7.780	< 0.001	
Model 3	Constant	85.313	9.126	0 < 0.001	0.106
	Birth Weight SDS	1.134	1.102	0.306	
	FFMI (kg/m^2^)	1.778	0.677	0.010	
DBP (mmHg)					
Model 1	Constant	78.981	4.606	< 0.001	0.032
	Birth Weight SDS	−0.146	0.845	0.863	
	Ln FMI (kg/m^2^)	−3.431	2.113	0.108	
Model 2	Constant	104.76	28.09	< 0.001	0.020
	Birth Weight SDS	−0.060	0.880	0.950	
	Ln Waist Circumference (cm)	−7.850	6.650	0.240	
Model 3	Constant	93.199	6.877	< 0.001	0.102
	Birth Weight SDS	0.341	0.830	0.682	
	FFMI (kg/m^2^)	−1.611	0.510	0.002	

*β* change in the outcome resulting from a one-unit change in the predictor value, *SE* Standard Error, *p* significance < 0.05, *R*^*2*^ % of variance explained by the model.

## Discussion

This study was set out to test the hypothesis that maternal investment in utero would shape the life history pace of the offspring. An accelerated life history pace (assessed by earlier menarcheal age) would cause the individual to prioritize reproduction overgrowth and homeostatic maintenance. In other words, the individual develops higher energy stores for reproductive purposes, and present with reduced growth and increased risk for chronic disease through poorer BP regulation, lower FFMI and higher central adiposity. In a sample of 94 young Emirati women (17–27 years), our results showed that birth weight SDS was positively and significantly associated with both height and leg length, while age at menarche did not show the same relationship. Body fat was inversely associated with menarcheal age, but not waist circumference (*p* > 0.05). Birth weight SDS was positively and significantly associated with waist circumference, FMI, and FFMI. All indices of adult adiposity (FMI and waist circumference) and FFMI were positively and significantly associated with SBP. Regarding DBP, the relationship was negatively and significantly associated with FFMI only.

Our data showed an inverse yet not significant association between birth weight and age at menarche, this did not reach significance; a finding which does not align with findings of some other studies [5, 37, 54]. An explanation given to this is that those born with lower birth weights present with higher amounts of adrenal concentrations in circulation, which results in earlier attainment of menarche [55]. However, these findings remain inconsistent across studies, as the opposite association has been demonstrated as well; that those born larger experience earlier menarche [56]. An alternative explanation offered for this phenomenon is that both elevated insulin-like growth factor-1 and leptin levels associated with a higher birth weight could trigger earlier menarche [55, 56]. Lack of observed significance in our study could also be attributed to failure in incorporating other factors that could affect age at menarche such as length at birth and postnatal growth rate. The suggested theoretical framework is not upheld in this population, which provokes further research into the specific environments and circumstances in which the relationship between birth weight and age at menarche is established.

A study in the Philippines concluded that menarcheal age was not significantly associated with birth weight alone, however ‘girls who were relatively long and thin at birth attained menarche approximately 6 months earlier than did girls who were short and light’ [57]. Recent research by Wells et al., in 2019, presented a theoretical model derived from life history theory where the researchers hypothesized that with lower maternal investment, early offspring reproduction was favored, body immune function was elevated, and risk-prone behaviors were increased. Results showed that daughters with early first reproduction had ‘growth trajectory favoring weight gain at the expense of linear growth’ [58]. This indicates that more markers need to considered, other than just early menarche, before deriving definitive conclusions about indicators of fast life history strategy.

Moreover, our findings support the hypothesis stating that lower maternal investment (lower birth weight) is linked to reduction in growth (proxied by height and leg length). This finding is consistent with earlier studies that reported the effect of birth weight on adult height [5, 59]. This is an important conclusion as adult height has been demonstrated to be inversely and significantly linked to mortality due to coronary heart disease [59]. Lower leg length measurements, in specific, may relate to higher risk of fatness, coronary heart disease and diabetes [45]. Independent of any other indicator, in our analysis, for every 1 cm increase in leg length, waist circumference increased by 1.01 cm (*p* = 0.005). Leg length was not significantly predictive of FFMI, FMI, SBP, and DBP. Additionally, those born small for gestational age and achieving shorter height were found to be associated with elevated sympathetic nerve activity [60]. This is suggested to be the link connecting maternal investment (birth weight), adult height and risk of hypertension and CVD [61]; not excluding the aforementioned function of nutrition and its effect on prenatal and postnatal growth.

In our analysis, lower birth weight was not linked to elevated adult adiposity levels, in fact the data showed the opposite association: the higher the birth weight, the higher the adult central adiposity (waist circumference). On the other hand, an earlier menarche was found to be associated with higher adult adiposity markers when adjusted for age. Our results are consistent with other findings reported in the meta-analysis conducted by Prentice and Viner in 2013 [62]. However, it is unclear whether earlier menarche is due to 1) fast postnatal catch-up growth following retarded intrauterine growth in the presence of nutritional affluence (associated with higher adult adiposity), or 2) low birth weight induced an increase in body fat in later life, or 3) both [61]. It is well documented that body composition changes with age, where fat mass increases and muscle mass decreases [63]. Hence, age was accounted for in the adiposity regression models (waist circumference and FMI). The analysis showed that age inclusion did not affect the results. This could be explained by the fact that our age group is young, hence no major shifts in body composition is seen at this stage of life [64]. Knowing the physical activity level could have given a better interpretation to the findings of both fat mass and fat free mass.

Clinical and experimental studies revealed that IUGR is associated with a significantly higher incidence of metabolic, renal and CVD in adult life where ‘IUGR directly cause cardiovascular alterations independent of pre-existing metabolic disease’ [65]. It has been proposed that early growth restriction in the womb due to lower maternal investment has been linked to higher rates of CVD risk by altering homeostatic capacity for blood pressure regulation [66], as well as increasing insulinemia [67]. Falkner stated that SBP is lowered by 1–2 mmHg with every kg increase in birth weight [68]. Contrary to the latter and other findings [5, 69], in this study, birth weight was not found to be indicative of adult blood pressure homeostasis as none of the models showed a significant relationship. Worth noting that, in all the SBP models, for every 1 unit increase in the BW SDS predicted an increase of 1.1 to 1.3 mmHg in SBP. As for the DBP, there was a general drop with increases in birth weight.

However, indices of adult adiposity were found to be positively and highly significantly linked to SBP. This stresses the importance of ‘metabolic load’ and post-natal environmental stressors on the development of disease rather than just birth weight. It is also important to note that while some studies have supported the link between birth weight and increased blood pressure, these findings were inconsistent. A study looking into the association between intrauterine growth and obesity suggested that it could be more useful to assess weight at birth “relative to genetic potential, indicated by the size of the parents or by the individuals body height” [21]. This is due to the evidence that support the theory that failure to reach growth potential has a higher association with blood pressure than solely birth weight [32]. Therefore, models that include post-natal growth and take into account parental genetics would be important to gain a better understanding of links to blood pressure regulation.

Waist circumference was found to be positively associated with birth weight SDS. This indicates that higher birth size equates to higher adult central adiposity. Some studies had found inverse associations between birth weight and abdominal obesity in adulthood than not [16–18].

Size at birth was also significantly and positively associated with FFMI. To explain why those who are born smaller end up with less adult fat free tissue mass, it has been suggested that suboptimal nutrition in prenatal period would restrict insulin secretions by causing hypoglycemia and so an upsurge in protein breakdown and a reduction in protein accretion [38]. Another pathway through which poor intrauterine conditions could affect FFM in adulthood is by lowering circulating levels of insulin-like growth factor 1, which in turn could affect intrauterine muscle growth [38]. Kuh et al. [70] added that the number of muscle fibers established at birth is related to birth weight. So, low FFM in those born smaller reduces metabolic capacity in adulthood and therefore increases disease risk especially in those who are subjected to an affluent environment [31].

It is important to note that although this type of study may offer us a chance to explore the links between maternal influences in prenatal period and adult disease risk, the issue remains much more multifaceted and there exist numerous limitations that make interpretation more complex. It is difficult to determine whether the heightened risk was due to lack of maternal investment and not attributable to other influences.

There were several limitations to the study. First, our study was cross-sectional, hence the inability to make causal relationship between early life events and their effect on later life outcomes [71]. Second, a relatively small sample size using a convenient sampling strategy was recruited, therefore impacting the generalizability of our results. Third, self-reporting was used to obtain participant information such as birth weight, gestational age, age at menarche, and breastfeeding duration. Our inability to verify this information by any other means such as hospital records meant that there is a possibility of recall bias due to memory limitations, which potentially threatens the internal validity of the study [72]. Also, recall might bias the mean measurements, reducing the variance and therefore probably masking relationships between variables. Fourth, there are many confounding factors that we did not account for and could have affected the study outcomes in terms of weight and body composition changes later in life or prenatal development, including postnatal circumstances such as dietary intake, physical activity levels and stress [5, 21]; maternal smoking habits [21, 73], and maternal blood pressure [13]. Finally, although socioeconomic data [74] was not collected, the UAE is a high-income country [75], and public universities are tuition-free for UAE nationals. Hence, it is safe to generalize that the sample population’s socioeconomic status would be representative of that of the national population. Overall, we consider this study relatively preliminary and encourage replication.

## Conclusion

This is a pioneer study in the UAE that sheds the light on the link between the intrauterine environment and potential disease risk. Although the study did not provide evidence for the proposed link between degree of maternal investment and menarcheal age, predictors of low maternal investment resulted in shorter height in adulthood. Earlier maturation was linked to higher adult adiposity but was not indicative of fat distribution. As for CVD risk, elevated blood pressure and adult central adiposity were linked to higher birth weight and/or gestational age, negating the hypothesis that weak maternal investment would result in hypertension. On the other hand, those born small were found to have lower FFM, which is indicative of higher CVD risk through possible interaction with glucose uptake mechanisms. Higher adult adiposity was also found indicative of higher SBP. This reiterates the role a higher metabolic load has on risk of disease. Over the past years, the UAE has witnessed developmental leaps and considerable changes in different life aspects naming health, economy, industry, education and most importantly lifestyle and eating habits [76]. What makes this research novel is the study population and the lifestyle changes that they are exposed to as a result of modernization and globalization. Hence, it would be interesting to examine our study hypotheses and assess the extent of how modernization impacts future mothers and their offspring. Moreover, in order to halt the epidemic of rising CVD diseases, the pathogenesis of the disease needs to be addressed. The idea that disease originates so early in human life can further our understanding and influence action. Health policies focusing on improving maternal nutrition during pregnancy are needed in order to prevent intrauterine malnutrition. This would help relieve not only the burden of morbidity and associated mortality but also lessen the massive economic burden that arises with chronic diseases.

## Supplementary Information

**Additional file 1.** Questionnaire and data collection form.

## Data Availability

Materials described in the manuscript, including all relevant raw data, will be freely available to any scientist wishing to use them for non-commercial purposes, without breaching participant confidentiality. The datasets used are available from the corresponding author.

## Abbreviations

AHA: American Heart Association
BMI: Body Mass Index
BP: Blood Pressure
CVD: Cardiovascular disease
DBP: Diastolic Blood Pressure
DXA: Dual Energy X-ray Absorptiometry
FFM: Fat Free Mass
FFMI: Fat Free Mass Index
FMI: Fat Mass Index
IUGR: Intrauterine Growth Restriction
SBP: Systolic Blood Pressure
UAE: United Arab Emirates
WHO: World Health Organization

## References

[1] Cardiovascular diseases (CVDs). Who.int. 2020 [cited June 2020]. Available from: https://www.who.int/news-room/fact-sheets/detail/cardiovascular-diseases-(cvds).

[2] United Arab Emirates. World Health Organization. 2020 [cited 12 June 2020]. Available from: https://www.who.int/countries/are/en/

[3] Dokken B (2008). The pathophysiology of cardiovascular disease and diabetes: beyond blood pressure and lipids. Diab Spectrum.

[4] Iacobellis G. Obesity and atherosclerosis. Obes Cardiovasc Dis. 2009:91–124. 10.1093/med/9780199549320.003.0007.

[5] Wells J, Yao P, Williams J, Gayner R (2016). Maternal investment, life-history strategy of the offspring and adult chronic disease risk in south Asian women in the UK. Evol Med Public Health.

[6] Tamosiunas A, Luksiene D, Baceviciene M, Bernotiene G, Radisauskas R, Malinauskiene V, Kranciukaite-Butylkiniene D, Virviciute D, Peasey A, Bobak M (2014). Health factors and risk of all-cause, cardiovascular, and coronary heart disease mortality: findings from the MONICA and HAPIEE studies in Lithuania. PLoS One.

[7] Hypertension. World Health Organization. World Health Organization; [cited 2021Mar22]. Available from: https://www.who.int/news-room/fact-sheets/detail/hypertension

[8] Wright JT, Williamson JD, Whelton PK, Snyder JK, Sink KM, Rocco MV, Reboussin DM, Rahman M, Oparil S, Lewis CE, Kimmel PL, Johnson KC, Goff DC, Fine LJ, Cutler JA, Cushman WC, Cheung AK, Ambrosius WT, SPRINT Research Group (2015). A randomized trial of intensive versus standard blood-pressure control. N Engl J Med.

[9] Mañalich R, Reyes L, Herrera M, Melendi C, Fundora I (2000). Relationship between weight at birth and the number and size of renal glomeruli in humans: a histomorphometric study. Kidney Int.

[10] Merlet-B Nichou C, Gilbert T, Muffat-Joly M, Leli Vre-P Gorier M, Leroy B (1994). Intrauterine growth retardation leads to a permanent nephron deficit in the rat. Pediatr Nephrol.

[11] Kopec G, Shekhawat P, Mhanna M (2017). Prevalence of diabetes and obesity in association with prematurity and growth restriction. Diab Metab Syndrome Obes.

[12] Chen W, Srinivasan S, Yao L, Li S, Dasmahapatra P, Fernandez C (2012). Low birth weight is associated with higher blood pressure variability from childhood to young adulthood: the Bogalusa heart study. Am J Epidemiol.

[13] Aye CY, Lewandowski AJ, Lamata P, Upton R, Davis E, Ohuma EO (2020). Prenatal and Postnatal Cardiac Development in Offspring of Hypertensive Pregnancies. J Am Heart Assoc.

[14] Després J (2012). Body fat distribution and risk of cardiovascular disease. Circulation..

[15] Klein S, Allison DB, Heymsfield SB, Kelley DE, Leibel RL, Nonas C, Kahn R (2007). Waist circumference and Cardiometabolic risk: a consensus statement from shaping America’s health: Association for Weight Management and Obesity Prevention; NAASO, the Obesity Society; the American Society for Nutrition; and the American Diabetes Association*. Obesity..

[16] Terry M, Wei Y, Esserman D (2007). Maternal, birth, and early-life influences on adult body size in women. Am J Epidemiol.

[17] Kuh D, Hardy R, Chaturvedi N, Wadsworth M (2002). Birth weight, childhood growth and abdominal obesity in adult life. Int J Obes.

[18] Law CM, Barker DJ, Osmond C, Fall CH, Simmonds SJ (1992). Early growth and abdominal fatness in adult life. J Epidemiol Community Health.

[19] Arnold LW, Hoy WE, Wang Z (2015). Low birth weight and large adult waist circumference increase the risk of cardiovascular disease in remote indigenous Australians — an 18year cohort study. Int J Cardiol.

[20] Boscaini C, Pellanda LC (2015). Birth weight, current anthropometric markers, and high sensitivity C-reactive protein in Brazilian school children. J Obes.

[21] Parsons TJ, Power C, Manor O (2001). Fetal and early life growth and body mass index from birth to early adulthood in 1958 British cohort: longitudinal study. BMJ..

[22] Rockenbach G, Luft VC, Mueller NT, Duncan BB, Stein MC, Vigo Á, Matos SMA, Fonseca MJM, Barreto SM, Benseñor IM, Appel LJ, Schmidt MI (2016). Sex-specific associations of birth weight with measures of adiposity in mid-to-late adulthood: the Brazilian longitudinal study of adult health (ELSA-Brasil). Int J Obes.

[23] Kathiresan S, Srivastava D (2012). Genetics of human cardiovascular disease. Cell..

[24] Jarvelin M-R (2000). Congenital heart disease: fetal and infant markers of adult heart diseases. Heart..

[25] Beal SJ, Hillman J, Dorn LD, Out D, Pabst S (2014). Associations between the prenatal environment and cardiovascular risk factors in adolescent girls: internalizing and externalizing behavior symptoms as mediators. Children’s Health Care.

[26] Hales CN, Barker DJP (2001). The thrifty phenotype hypothesis. Br Med Bull.

[27] Forsdahl A (1977). Are poor living conditions in childhood and adolescence an important risk factor for arteriosclerotic heart disease?. J Epidemiol Community Health.

[28] Calkins K, Devaskar SU (2011). Fetal origins of adult disease. Curr Problems Pediatr Adolesc Health Care.

[29] Godfrey KM, Lillycrop KA, Burdge GC, Gluckman PD, Hanson MA (2007). Epigenetic Mechanisms and the Mismatch Concept of the Developmental Origins of Health and Disease. Pediatr Res.

[30] Wells JC (2011). The thrifty phenotype: an adaptation in growth or metabolism?. Am J Hum Biol.

[31] Wells JC (2010). Maternal capital and the metabolic ghetto: an evolutionary perspective on the transgenerational basis of health inequalities. Am J Hum Biol.

[32] Leon DA, Koupilova I, Lithell HO, Berglund L, Mohsen R, Vagero D, Lithell UB, McKeigue PM (1996). Failure to realise growth potential in utero and adult obesity in relation to blood pressure in 50 year old Swedish men. Bmj..

[33] Giudice MD, Gangestad SW, Kaplan HS (2015). Life history theory and evolutionary psychology. Handbook Evol Psychol.

[34] Kuzawa CW (2004). Fetal origins of developmental plasticity: are fetal cues reliable predictors of future nutritional environments?. Am J Hum Biol.

[35] Wells J (2018). Life history trade-offs and the partitioning of maternal investment. Evol Med Public Health.

[36] Bleil ME, Adler NE, Appelhans BM, Gregorich SE, Sternfeld B, Cedars MI (2013). Childhood adversity and pubertal timing: understanding the origins of adulthood cardiovascular risk. Biol Psychol.

[37] Proos LA (2009). Growth and development of Indian children adopted in Sweden. Indian J Med Res.

[38] Singhal A, Wells J, Cole TJ, Fewtrell M, Lucas A (2003). Programming of lean body mass: a link between birth weight, obesity, and cardiovascular disease?. Am J Clin Nutr.

[39] Eriksson J, Forsén T, Tuomilehto J, Osmond C, Barker D (2002). Size at birth, fat-free mass and resting metabolic rate in adult life. Horm Metab Res.

[40] Aihie Sayer A, Syddall HE, Dennison EM, Gilbody HJ, Duggleby SL, Cooper C, Barker DJ, Phillips DI (2004). Birth weight, weight at 1 y of age, and body composition in older men: findings from the Hertfordshire cohort study. Am J Clin Nutr.

[41] Evans PL, McMillin SL, Weyrauch LA, Witczak CA (2019). Regulation of skeletal muscle glucose transport and glucose metabolism by exercise training. Nutrients..

[42] Srikanthan P, Karlamangla AS (2011). Relative muscle mass is inversely associated with insulin resistance and prediabetes. Findings from the third National Health and nutrition examination survey. J Clin Endocrinol Metab.

[43] Buyken A, Karaolis-Danckert N, Remer T, Bolzenius K, Landsberg B, Kroke A (2008). Effects of breastfeeding on trajectories of body fat and BMI throughout childhood. Obesity..

[44] Montagnese C, Nutile T, Marphatia AA, Grijalva-Eternod CS, Siervo M, Ciullo M, Wells JC (2014). Body composition, leg length and blood pressure in a rural Italian population: a test of the capacity-load model. Nutr Metab Cardiovasc Dis.

[45] Bogin B, Varela-Silva M (2010). Leg length, body proportion, and health: a review with a note on beauty. Int J Environ Res Public Health.

[46] Said-Mohamed R, Prioreschi A, Nyati L, van Heerden A, Munthali R, Kahn K (2018). Rural–urban variations in age at menarche, adult height, leg-length and abdominal adiposity in black south African women in transitioning South Africa. Ann Hum Biol.

[47] Waist circumference and waist-hip ratio: report of a WHO expert consultation. World Health Organization. World Health Organization; 2011 [cited 2021 Mar21]. Available from: https://www.who.int/publications/i/item/9789241501491

[48] Body mass index - BMI. World Health Organization. World Health Organization; 2021 [cited 2021Mar21]. Available from: https://www.euro.who.int/en/health-topics/disease-prevention/nutrition/a-healthy-lifestyle/body-mass-index-bmi

[49] Litwin SE (2008). Which measures of obesity best predict cardiovascular risk?. J Am Coll Cardiol.

[50] Liu P, Ma F, Lou H, Liu Y (2013). The utility of fat mass index vs. body mass index and percentage of body fat in the screening of metabolic syndrome. BMC Public Health.

[51] Vanitallie TB, Yang MU, Heymsfield SB, Funk RC, Boileau RA (1990). Height-normalized indices of the body’s fat-free mass and fat mass: potentially useful indicators of nutritional status. Am J Clin Nutr.

[52] High Blood Pressure. www.heart.org. [cited 2021Mar21]. Available from: https://www.heart.org/en/health-topics/high-blood-pressure

[53] Fenton TR, Kim JH (2013). A systematic review and meta-analysis to revise the Fenton growth chart for preterm infants. BMC Pediatr.

[54] Ghirri P, Bernardini M, Vuerich M, Cuttano AM, Coccoli L, Merusi I (2001). Adrenarche, pubertal development, age at menarche and final height of full-term, born small for gestational age (SGA) girls. Gynecol Endocrinol.

[55] Zhang Z, Hartman TJ (2013). Birth weight is associated with age at menarche in US girls. Clin Pediatr.

[56] Terry MB, Ferris JS, Tehranifar P, Wei Y, Flom JD (2009). Birth weight, postnatal growth, and age at menarche. Am J Epidemiol.

[57] Adair LS (2001). Size at Birth Predicts Age at Menarche. Pediatrics.

[58] Wells J, Cole T, Cortina-Borja M, Sear R, Leon D, Marphatia A (2019). Low maternal capital predicts life history trade-offs in daughters: why adverse outcomes cluster in individuals. Front Public Health.

[59] Sorensen HT, Sabroe S, Rothman KJ, Gillman M, Steffensen FH, Fischer P, Serensen TIA (1999). Birth weight and length as predictors for adult height. Am J Epidemiol.

[60] Boguszewski MC, Johannsson G, Fortes LC, Sverrisdóttir YB (2004). Low birth size and final height predict high sympathetic nerve activity in adulthood. J Hypertens.

[61] Tam CS, Zegher FD, Garnett SP, Baur LA, Cowell CT (2006). Opposing influences of prenatal and postnatal growth on the timing of menarche. J Clin Endocrinol Metab.

[62] Prentice P, Viner RM (2012). Pubertal timing and adult obesity and cardiometabolic risk in women and men: a systematic review and meta-analysis. Int J Obes.

[63] St-Onge M-P, Gallagher D (2010). Body composition changes with aging: the cause or the result of alterations in metabolic rate and macronutrient oxidation?. Nutrition..

[64] Siervogel R, Wisemandle W, Maynard L, Guo S, Roche A, Chumlea W (1998). Serial changes in body composition throughout adulthood and their relationships to changes in lipid and lipoprotein levels. Arterioscler Thromb Vasc Biol.

[65] Menendez-Castro C, Rascher W, Hartner A (2018). Intrauterine growth restriction - impact on cardiovascular diseases later in life. Mol Cell Pediatr.

[66] Barker DJP (1998). In utero programming of chronic disease. Clin Sci.

[67] Jaquet D, Deghmoun S, Chevenne D, Collin D, Czernichow P, Lévy-Marchal C (2005). Dynamic change in adiposity from fetal to postnatal life is involved in the metabolic syndrome associated with reduced fetal growth. Diabetologia..

[68] Falkner B (2002). Birth weight as a predictor of future hypertension. Am J Hypertens.

[69] Al Salmi I, FA MS, Hannawi S (2019). Birth weight, gestational age, and blood pressure: Early life management strategy and population health perspective. Saudi J Kidney Dis Transpl.

[70] Kuh D, Bassey J, Hardy R, Aihie Sayer A, Wadsworth M, Cooper C (2002). Birth weight, childhood size, and muscle strength in adult life: evidence from a birth cohort study. Am J Epidemiol.

[71] Levin K (2006). Study design III: cross-sectional studies. Evid-Based Dentistry.

[72] Hassan E (2005). Recall bias can be a threat to retrospective and prospective research designs. Int J Epidemiol.

[73] Wehby G, Prater K, McCarthy A, Castilla E, Murray J (2011). The impact of maternal smoking during pregnancy on early child neurodevelopment. J Hum Cap.

[74] Forman MR, Mangini LD, Thelus-Jean R, Hayward MD (2013). Life-course origins of the ages at menarche and menopause. Adolesc Health Med Ther.

[75] Data.worldbank.org. 2021. United Arab Emirates | Data. Available at: <https://data.worldbank.org/country/AE> [Accessed 9 May 2021].

[76] Sulaiman N, Hussein A, Saddik B, Elbadawi S, Hasswan A, Emad Z, Mahmoud I (2020). Community health perceptions of smoking, physical activity and eating habits: a cross-sectional, descriptive study. Hamdan Med J.

