# Supplementary material for: Maternal investment, life-history trajectory of the off-spring and cardiovascular disease risk in Emirati females in the United Arab Emirates
Source: BMC Public Health. 2021 Jun 27;21:1237. doi: 10.1186/s12889-021-11182-0 (PMC8237435; doi:10.1186/s12889-021-11182-0)
Supplement: Supplementary file 1 — Additional file 1. Questionnaire and data collection form. [file 12889_2021_11182_MOESM1_ESM.docx]

**Assessments Checklist**

| - **Questionnaire** |
| --- |
| - **Physical Activity** |
| - **Anthropometric Measurements** |
| - **Blood Pressure** |
| - **Tanita** |
| - **InBody** |
| - **Indirect Calorimetry** |
| - **BodPod** |
| - **iDEXA** |
| - **Grip Strength** |
| - **24 hour recall** |

**Questionnaire**

| Telephone |  |
| --- | --- |
| Email |  |
| Date of birth |  |
| Age |  |

| College: | __: ABP |
| --- | --- |
|  | __: College of Art and Creative Enterprises |
|  | __: College of Business |
|  | __: College of Communication & Media Sciences |
|  | __: College of Technological Innovation |
|  | __: College of Sustainability Sciences and Humanities |
|  | __: University college |
| Have you ever taken or are you taking any Nutrition courses? | __: Yes __:No |

| Where are you from? | Emirati: __ | Other (please specify): __ |
| --- | --- | --- |
| Were you born and raised in the UAE? | __: Yes | __: No |
| If answer is no, provide details of residence country. |  | |

| Do you smoke? | __: Yes | | __: No | |
| --- | --- | --- | --- | --- |
| *Smoking Status (if yes):* | | | | |
| Cigarettes per day | Light smoker (less than 10 cigarettes) | Moderate smoker (10-19 cigarettes) | | Heavy smoker (more than 20 cigarettes) |
| Shisha/Hooka per day | Light smoker (less than 15 minutes of shisha) | Moderate smoker (30 minutes of shisha) | | Heavy smoker (one hour of shisha) |

| Are you married? | __: Yes | | | __: No |
| --- | --- | --- | --- | --- |
| Are you currently pregnant | __: Yes | | | __: No |
| Do you have children? | __: Yes | | | __: No |
| *If yes, to previous question?* | | | | |
| *Child* | *Age* | *Sex* | *Did you breastfeed?* | *If yes, duration:* |
| *1.* |  |  |  |  |
| *2.* |  |  |  |  |
| *3.* |  |  |  |  |
| *4.* |  |  |  |  |
| *5.* |  |  |  |  |

| Date of the first day of last menstrual day |  |
| --- | --- |
| Number of days in a whole menstrual cycle |  |

Example: 5 days menstruation then 25 days to next menstruation= 30 day cycle

| Age at menarche (age at which you first got your period) |  |
| --- | --- |
| Your weight at birth (kg) |  |
| Were you breastfed? If yes, for how long? | ___:Yes, Duration: ______ _____:No |
| What is your birth order? |  |
| How many siblings do you have, if any including age and gender. | Sibling 1: M/F Age:  Sibling 2: M/F Age:  Sibling 3: M/F Age:  Sibling 4: M/F Age:  Sibling 5: M/F Age:  Sibling 6: M/F Age: |
| Your gestational age (in weeks). |  |
| Are you a twin? | __: Yes __:No |

| Weight of the mother at time of your birth |  |
| --- | --- |
| Mother’s current weight |  |
| Mother’s height |  |
| Mother’s age at your birth |  |
| Mother’s age at menarche (age she got her period) |  |

Do you have any of the following diseases?

__: Hypertension

__: Diabetes

__: Heart Problems

__: Asthma

__: Thyroid gland related condition

Other (please specify): ___________

| Are you taking any medication? | | __: Yes | | __: No |
| --- | --- | --- | --- | --- |
| If your answer is yes, what kind of medication are you taking?  Type:______________________________________Dosage:_______________________  Type:______________________________________Dosage:_______________________ | | | | |
| Are you taking any supplements? | __: Yes | | __: No | |
| If your answer is yes, what kind of supplement you are taking?  Type:______________________ Dosage:______________________________ Type:______________________ Dosage:______________________________  Other (please specify):_________________________________________________________________ | | | | |

| Do you have a pacemaker | __: Yes | __: No |
| --- | --- | --- |

| Have you ever had any surgery? | | | __: Yes | | __: No |
| --- | --- | --- | --- | --- | --- |
| __: Lung | __: Heart | __: Liver | | __: Kidney | |
| Other (please specify):___________________________________________________ | | | | | |

| Have you ever had any broken bones? | __: Yes | __: No |
| --- | --- | --- |
| If yes: Location: _______________________________  Cause: _________________________________  Age: ___________________________________ | | |

| Over the last 3 months, has your weight changed? | __: Yes | __: No |
| --- | --- | --- |
| If yes, by how many Kg? ________ | | |

**Data Collection Sheet**

**A) Physical activity**

| Level of physical activity | __: Sedentary (no exercise) |
| --- | --- |
|  | __: Low active (1-3 days a week) |
|  | __: Active (4-5 days a week) |
|  | __: Moderate active (6-7 days a week) |
|  | __: Highly active (twice per day extra heavy workout) |

**B) Anthropometrics measurement**

| Weight From Bod Pod machine (Kg) |  |
| --- | --- |
| Height (cm) |  |
| Sitting height (cm) |  |
| Waist circumference (cm) |  |
| Hip circumference (cm) |  |
| Calf circumference (cm) |  |
| Mid-arm circumference (cm) |  |

**C) Blood pressure**

| Blood pressure | Systolic | Diastolic | Heart rate |
| --- | --- | --- | --- |
|  |  |  |  |
|  |  |  |  |
|  |  |  |  |

**D) Bioelectrical Impendence**

| **Tanita** | |
| --- | --- |
| % Fat |  |
| Fat mass (kg) |  |
| Fat free mass (kg) |  |
| Resting metabolic rate |  |
| Resistance |  |

| **InBody** | |
| --- | --- |
| % Fat |  |
| Fat mass (kg) |  |
| Fat free mass (kg) |  |
| Resting metabolic rate |  |
| Waist to hip ratio |  |

**E) BodPod**

| %Fat (kg) |  |
| --- | --- |
| Fat mas (kg) |  |
| Fat free mass (kg) |  |
| Body Density |  |
| Body volume |  |
| Resting metabolic rate |  |

**F) Indirect Calorimetry**

| Measured resting metabolic rate (RMR) |  |
| --- | --- |

**G) iDEXA**

| **Total Body** | |
| --- | --- |
| Bone mineral content |  |
| Bone mineral density |  |
| % Fat (kg) |  |
| Fat mas (kg) |  |
| Fat free mass (kg) |  |

**24 hour recall sheet (weekday)**

| Meal | Food/drink |
| --- | --- |
| Breakfast |  |
| Snack |  |
| Lunch |  |
| Snack |  |
| Dinner |  |
| Snack |  |
